# Effects of Individualized Gait Rehabilitation Robotics for Gait Training on Hemiplegic Patients: Before-After Study in the Same Person

**DOI:** 10.3389/fnbot.2021.817446

**Published:** 2022-03-08

**Authors:** Zhao Guo, Jing Ye, Shisheng Zhang, Lanshuai Xu, Gong Chen, Xiao Guan, Yongqiang Li, Zhimian Zhang

**Affiliations:** ^1^School of Power and Mechanical Engineering, Wuhan University, Wuhan, China; ^2^Shenzhen Milebot Robotics Co., Ltd., Shenzhen, China; ^3^School of Mechanical Engineering, Shenyang Jianzhu University, Shenyang, China; ^4^Department of Health Management Center, Qilu Hospital, Shandong, China; ^5^Rehabilitation Medicine Center, The First Affiliated Hospital, Nanjing Medical University, Nanjing, China; ^6^The Cheeloo College of Medicine, Shandong University, Jinan, China

**Keywords:** individualized gait, hemiplegic patient, gait rehabilitation, exoskeleton robot, stroke

## Abstract

**Background:**

Lower-limb exoskeleton robots are being widely used in gait rehabilitation training for patients with stroke. However, most of the current rehabilitation robots are guided by predestined gait trajectories, which are often different from the actual gait trajectories of specific patients. One solution is to train patients using individualized gait trajectories generated from the physical parameters of patients. Hence, we aimed to explore the effect of individual gaits on energy consumption situations during gait rehabilitation training for hemiplegic patients with lower-limb exoskeleton robots.

**Methods:**

A total of 9 unilateral-hemiplegic patients were recruited for a 2-day experiment. On the first day of the experiment, the 9 patients were guided by a lower-limb exoskeleton robot, walking on flat ground for 15 min in general gait trajectory, which was gained by clinical gait analysis (CGA) method. On the other day, the same 9 patients wore the identical robot and walked on the same flat ground for 15 min in an individualized gait trajectory. The main physiological parameters including heart rate (HR) and peripheral capillary oxygen saturation (SpO2) were acquired *via* cardio tachometer and oximeter before and after the walking training. The energy consumption situation was indicated by the variation of the value of HR and SpO2 after walking training compared to before.

**Results:**

Between-group comparison showed that the individualized gait trajectory training resulted in an increase in HR levels and a decrease in SpO2 levels compared to the general gait trajectory training. The resulting difference had a statistical significance of *p* < 0.05.

**Conclusion:**

Using individualized gait guidance in rehabilitation walking training can significantly improve energy efficiency for hemiplegic patients with stroke.

## Introduction

Globally, stroke, which is a major cause of limb functional disabilities, is the disease with the highest disability rate (80 ~ 90%). In recent years, the population of patients with stroke continues to grow at a rate of nearly 9% every year (Liu et al., 2007), and it shows a trend in the youth community. The disease is causing permanent serious harm to the patients and brings a heavy medical burden to patients' families and society.

In recent years, more and more attention has been paid to the application of exoskeleton robots in the field of rehabilitation, in particular neurorehabilitation. The rehabilitation robot, such as LOPES (Meuleman et al., 2015), Lokomat (Riener et al., 2010), WalkTrainer (Stauffer et al., 2008), ALEX (Banala et al., 2008), Indego (Hartigan et al., 2015), HAL (Tsukahara et al., 2014), ReWalk (Esquenazi et al., 2012), Ekso (Kozlowski et al., 2015), replace the rehabilitation physician to provide physical therapy to patients and to carry out safe and reliable repetitive training for patients, which help to reduce the workload of rehabilitation physician in physical therapy and improve the effectiveness of rehabilitation treatment (Meng et al., 2015).

In the above devices, a gait control strategy based on finite state or predetermined gait trajectory is adopted. These general gait trajectories of hip joint, knee joint, and ankle joint are the statistical results of many healthy people (J Robert Close, 1952; Murray et al., 1964; Johnston and Smidt, 1969). However, many studies have shown that physical factors—walking speed, gender, age, and other anthropometric parameters—led to different gait patterns in different groups (Wang et al., 2003; Kale et al., 2004). The existing wearable exoskeleton control strategy cannot meet individual differences for different users (Chen et al., 2016). In order to provide specific gait guidance for exoskeleton wearers, gait prediction has become a popular research branch (Zhang and Ma, 2019; Khera and Kumar, 2020). Vallery et al. (2008) proposed a complementary limb motion estimation algorithm, which can generate real-time trajectory to provide compensation for hemiplegic patients, but its goal is to achieve the symmetry between legs, rather than periodic gait sequence. Kagawa et al. (2015) proposed the method of motion planning control in joint space to provide variable step length and speed for exoskeleton, but the gait mode is not natural, because the limited fixed joint angle is predefined for trajectory planning. However, these studies lack clinical verification. Rajasekaran et al. (2018) applied a brain-computer interface to exoskeleton control and conducted clinical trials on 4 patients with spinal cord injury. However, when there is no auxiliary trajectory guidance, it is difficult for patients to walk normally after rehabilitation.

Our study aimed to examine the energy consumption effects of individualized gait trajectory in walking rehabilitation among nine patients with hemiplegic status-post stroke with the assistance of a lower-limb exoskeleton robot named BEAR-H1. This paper proposed a clinical metric for measuring a patient's energy consumption level after walking rehabilitation. Heart rate (HR) and peripheral capillary oxygen saturation (SpO2) are selected as the independent variables to reflect the energy level of walking guided by an individualized gait trajectory compared with that of walking guided by a general gait trajectory, which is a locomotion data from Clinical Gait Analysis (CGA).

We combined Fast Fourier Transformation (FFT) and Gaussian Process Regression (GPR) to generate individualized gait trajectories, which could be adjusted according to different patients. The proposed individualized gait trajectory generation algorithm was tested with the cross-validation method. The high accuracy and strong robustness of the algorithm were validated referring to the CGA data. The Mean Absolute Error (MAE) and the SD of predicted joint rotation angles of the individualized gait trajectories were optimized to the most extent. Finally, the algorithm was applied to a new lower extremity exoskeleton BEAR-H1 to train patients (Yun et al., 2014; Kong et al., 2018). Nine stroke patients with different morphological parameters were recruited for a clinical trial, which was helpful to observe the diversified behaviors of rehabilitation strategies.

Training results showed that compared to general gait trajectory, there was an increase in HR and a decrease in SpO2 when the robot was controlled by individualized gait trajectories. Specifically, changes in HR were more significant. On the contrary, changes in SpO2 were much smaller. This contrast indicated that the individualized gait strategy was energy friendly for hemiplegic patients.

## Exoskeleton BEAR-H1 Platform

**Figure 1:**
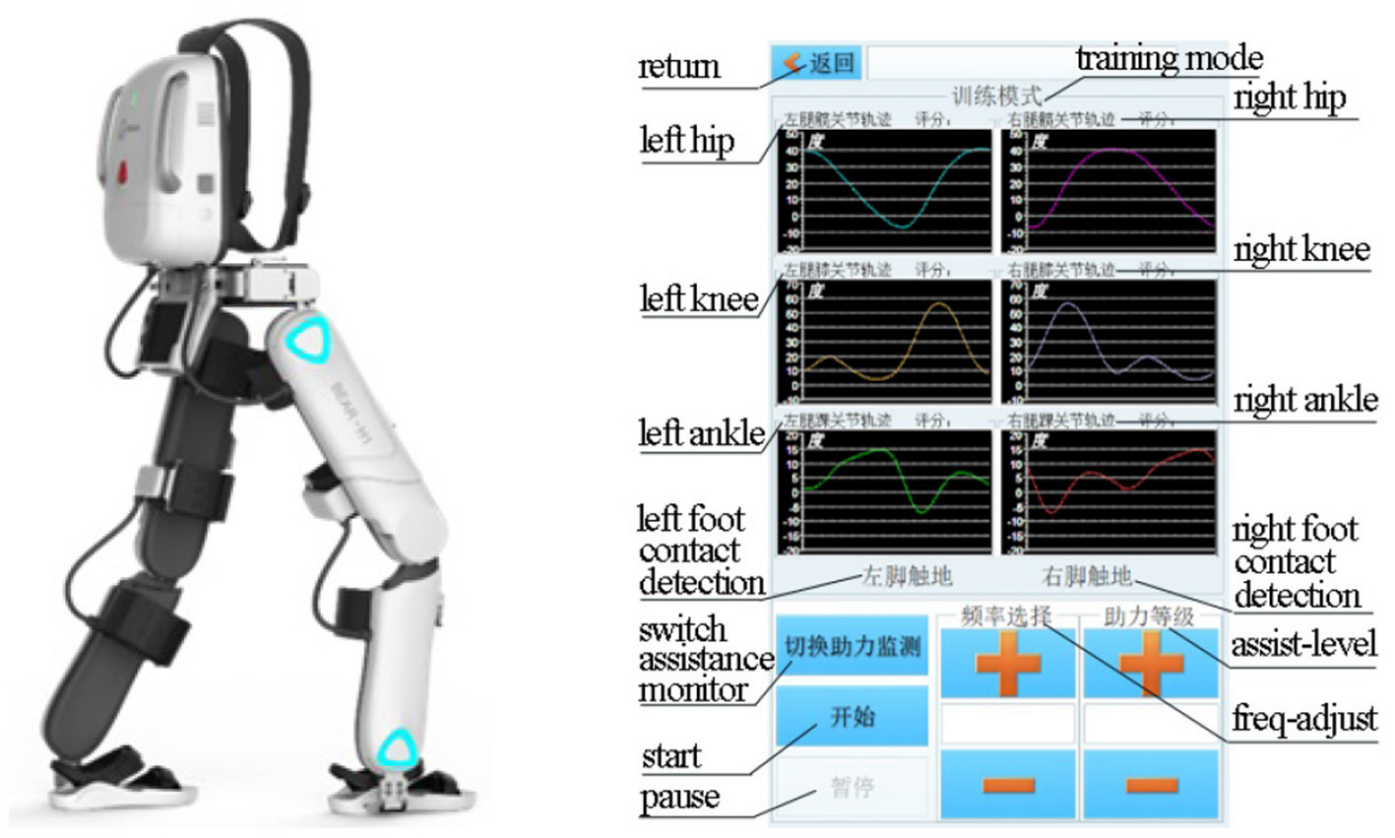
Left panel: BEAR-H1 robot; Right panel: gait trajectories.

BEAR-H1 is a wearable, battery-powered lower-limb rehabilitation robot with initiative assisting technology, and it enables gait events to be detected when the subjects are wearing the BEAR-H1, as shown in Figure 1 left panel. The robot, which has three active degrees of freedom and a passive degree of freedom on each leg, is self-developed to help patients with hemiplegia conduct rehabilitation training. The three degrees of freedom are rotations along the hip joint, the knee joint, and the ankle joint on the sagittal plane and they are actuated by motors (Santos et al., 2012). The adduction and abduction of the hip joint is the passive degree of freedom (Kotwicki et al., 2008). There is a rotary encoder in each joint of BEAR-H1, as shown in Figure 1 left panel, which is used to measure the real-time angle of each joint (Zhang et al., 2015). The actuator can accurately control the joint angle by the feedback of the encoder. The gait trajectories, as shown in Figure 1 right panel, can be changed easily by modifying the program of the robot.

For the purpose of the present study, we embedded different individualized gait trajectories that was corresponding to specific patient's training into the internal storage in the micro-controller unit in advance. The control process was executed at 1,000 Hz and the testing time for the patient wearing the BEAR-H1 was about 15 min. The level of assistance was variable according to the patient's actual walking ability level.

## Individualized Gait Reconstruction

**Figure 2:**
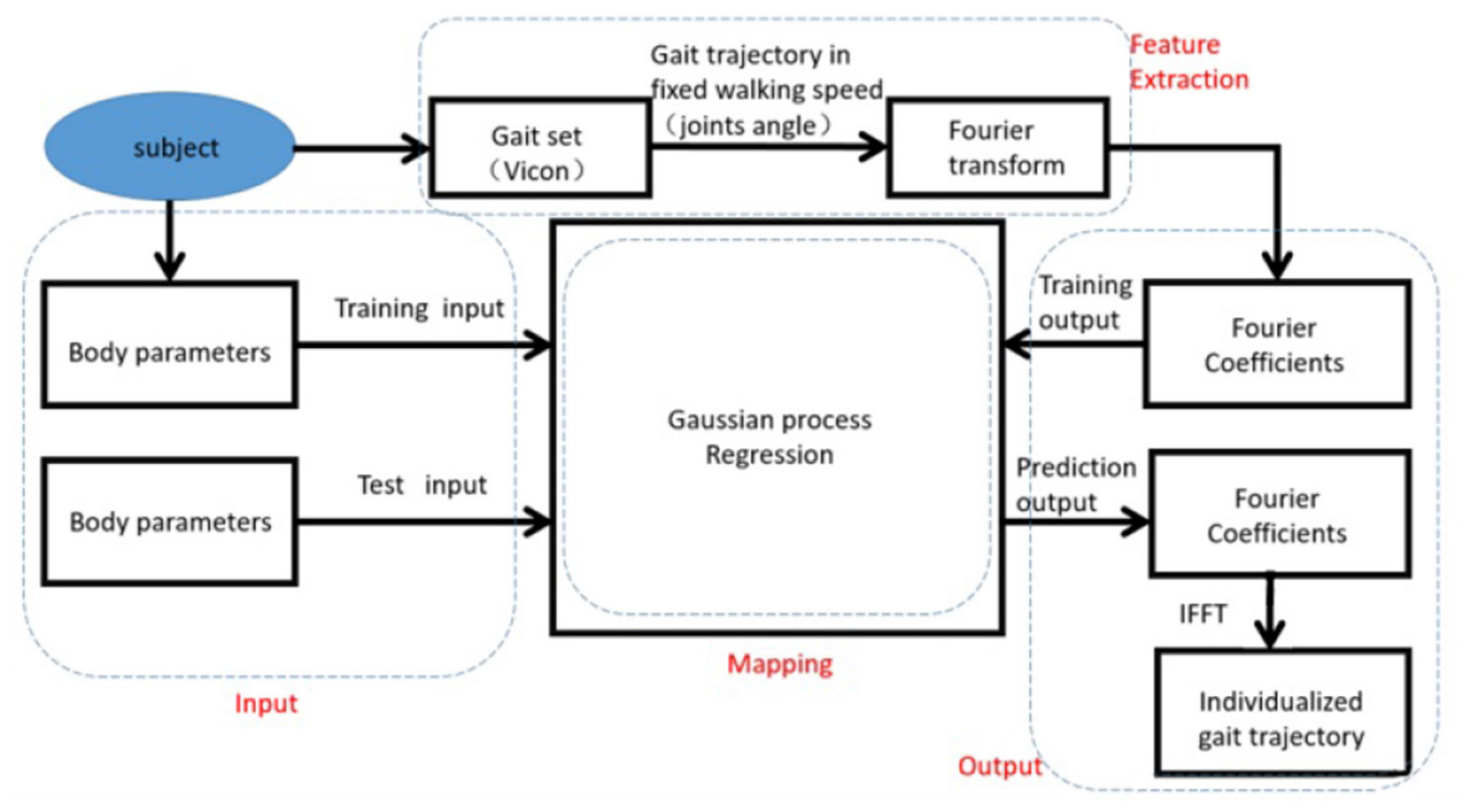
Outline of generation process, consist of four sections which are Input, Feature Extraction, Mapping, and Output.

The generation process for individualized gait reconstruction includes four components. As shown in Figure 2, the input component is consisted of body parameters only. Gait data is divided into various sets according to different waking speeds. A certain walking speed is selected, linking to a specific set for feature extraction. During the feature extraction, encoding progress employs an explicable model for apprehensible processing which is Fourier Transform. Correspondingly, decoding and reconstruction for generating the final individualized gait pattern are finished by Fourier Inversion at the output component.

In order to establish the mapping relationships between the body parameters and the gait pattern, the gait pattern is first extracted into Fourier Coefficients to reduce the computational cost from numerous data points to handful coefficients (Reddy and Rani, 2016). The Fourier Coefficients can be predicted through GPR with body parameters. Finally, the individualized gait pattern is reconstructed based on predictive Fourier Coefficients.

###  Gait Feature Extraction and Anthropometry

Gait patterns are represented as the trajectories of lower-limb joints, which are joints of hips, knees, and ankles (Isola et al., 2011). Although gait patterns determined various gait features, they are time-sequence signals with the periodic pattern (Trivino et al., 2010), which is the most often domain applied with Fourier Transform (Morgan and Noehren, 2018).

**Figure 3:**
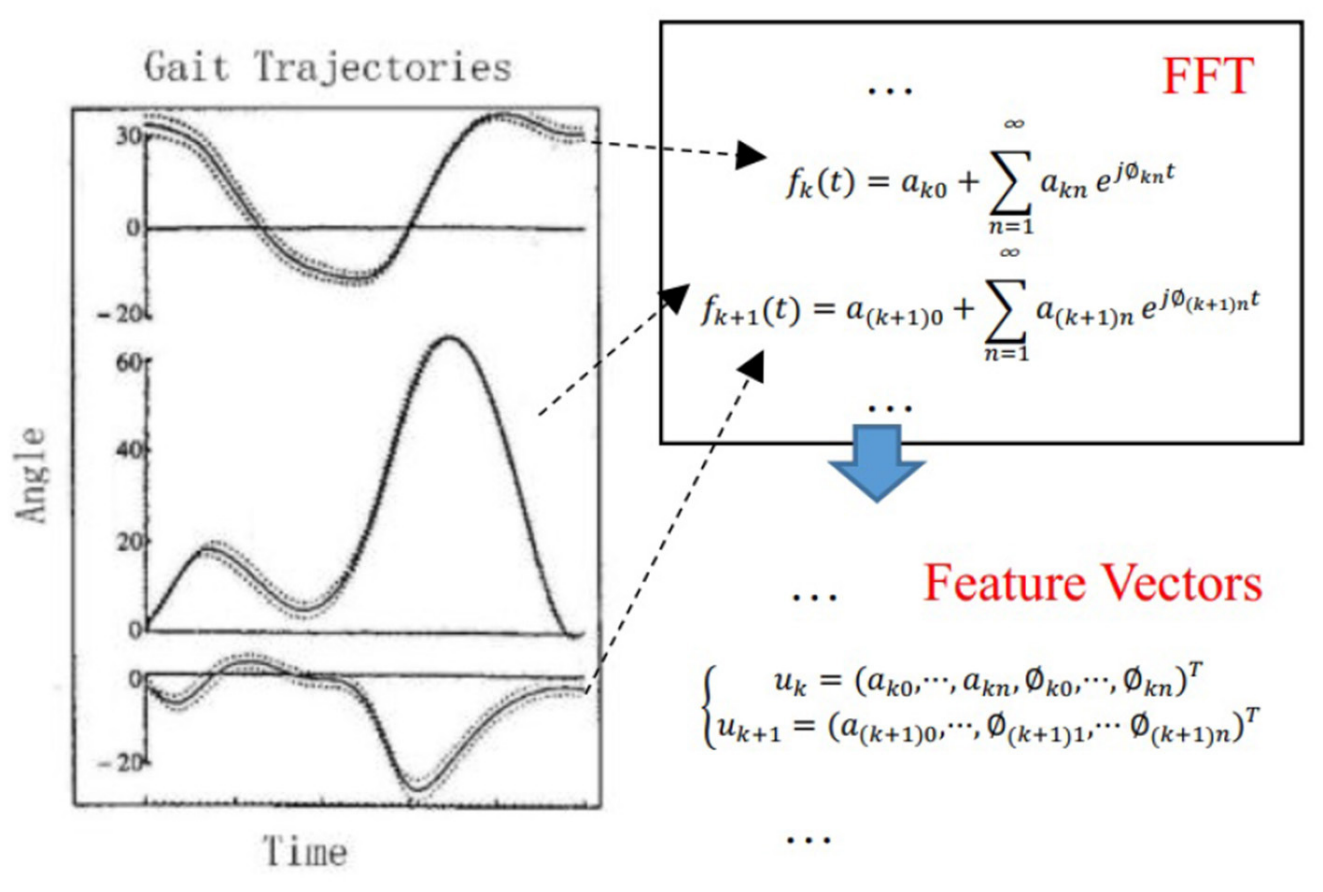
Feature extraction progress. Gait pattern of the rotation angle of left hip, left knee, left ankle, right hip, right knee, and right ankle are transformed into Fourier coefficients through Fast Fourier transform. The actual hip joint, knee joint, and ankle joint rotation angles were measured with angular encoders on the exoskeleton. The distribution of the actual angles was illustrated in the Figure. Regions that enclosed by dotted curves are the distribution of the actual angles of a healthy subject.

Fourier transform is a traditional spectral analysis method to describe any periodic signal in its harmonic components (Winter, 2009). Since walking is periodic and the power for walking is supplied rhythmically with temporal consistency (Winter, 2009), Fourier transform is often used to describe the frequency content of gait (Antonsson and Mann, 1985; Chau, 2001). In our study, each joint angle waveform was analyzed in the frequency domain and decomposed into one Fourier coefficient and frequency vector as the gait features:


(1) $$\begin{array}{l} \mu_{k}={\left(a_{k0},\cdot \cdot \cdot ,a_{kn},\phi_{k1},\cdot \cdot \cdot ,\phi_{kn}\right)}^{T} \end{array}$$

where *a*_*kn*_ is the Fourier coefficients, ϕ_*k*_*n* is the frequency of harmonic wave, and *k* is the number of walking trials. Note that ϕ_0_ = 0. In this paper, we take *n* = 3. Feature extraction progress is shown in Figure 3.

**Figure 4:**
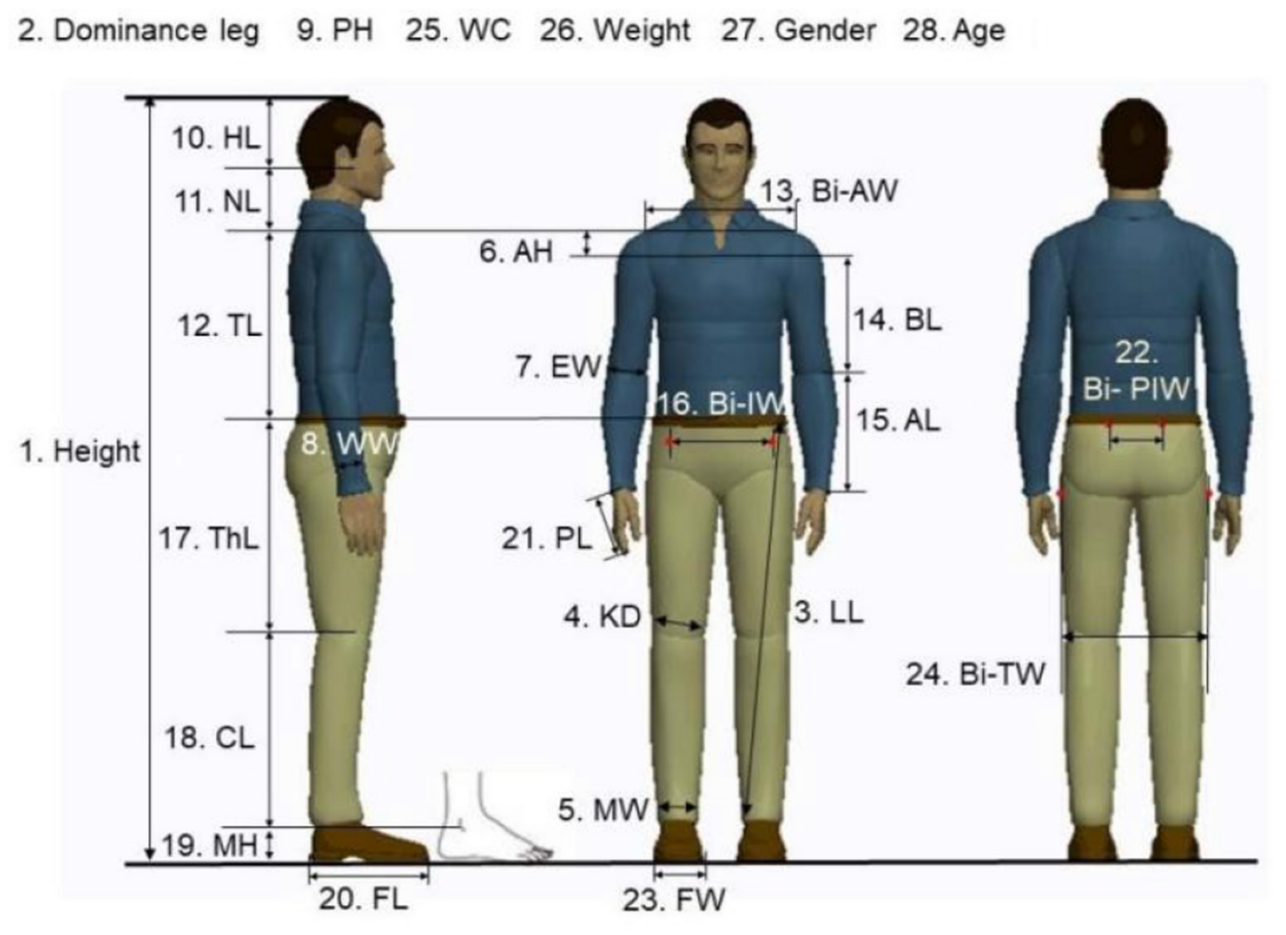
Gait related body parameters 3. LL: Leg Length; 4. KD: knee diameter; 5. MW: Malleolus width; 6. AH: Acromion height; 7. EW: Elbow width; 8. WW: Wrist width; 9. PH: Palm height; 10. HL: Head length; 11. NL: Neck length; 12. TL: Trunk length; 13. Bi-AW: Bi-acromion width; 14. BL: Brachium length;15. AL: Antebrachium length; 16. Bi-IW: Bi-iliac width; 17. ThL: Thigh length; 18. CL: Calf length; 19. MH: Malleolus height; 20. FL: Foot Length; 21. PL: Palm length; 22. Bi-PIW: Bi-posterior iliac; 23. FW: Foot width; 24. Bi-TW: Bi-trochanteric width; 25. WC: Waist circumference.

Gait patterns are determined by various factors. To fully study the influence of different parameters on the gait pattern, a total of 28 body parameters are considered in this paper, as shown in Figure 4. Then, the vector of body parameters for the *i*^*th*^ human subject can be formulated as.


(2) $$\begin{array}{l} B_{i}={\left(b_{1},\cdot \cdot \cdot ,b_{28}\right)}^{T} \end{array}$$

###  Gaussian Process Regression

In order to obtain the mapping relationship between each gait feature u and human body parameters B. We implemented the GPR algorithm for achieving our goal since gait feature prediction is regarded as a nonlinear regression task. As a kernel-based statistical learning method, GPR is with advantages for solving the small sample learning problem (Cen et al., 2021), which suits the scenario that limited human subjects are included in the database. A detailed description of GPR can be studied in Rasmussen and Williams (2006).

The performance of the proposed scheme can be assessed by comparing the difference between the generated gait and the actual gait of the subject (i.e., measured by the sensor), in terms of the correlation coefficient (3) and the mean absolute error (MAE) (4). A higher correlation coefficient between the predicted and actual and smaller values of MAE implies a better performance of the proposed scheme, and vice versa (Mukaka, 2012; Mundt et al., 2020).


(3) $$\begin{array}{l} \rho =\frac{cov\left(\tilde{\theta },\hat{\theta }\right)}{\sqrt{var\left(\tilde{\theta }\right)var\left(\hat{\theta }\right)}} \end{array}$$

(4) $$\begin{array}{l} e_{MAE}=\overset{L_{0}}{\underset{i=1}{\sum }}\frac{\left|{\hat{\theta }}_{i}-{\tilde{\theta }}_{i}\right|}{L_{0}} \end{array}$$

Where *L*_0_ is the fixed length to which the gait cycle is resampled to. ${\tilde{\theta }}_{i}$ is the *i*^*th*^ actual angle of joint after resampling. ${\hat{\theta }}_{i}$ is the *i*^*th*^ predicted angle of joint.

###  Algorithm Performance

The performance of the proposed algorithm was validated by the cross-validation method using the training set. Due to the limited data and to make full use of it, a leave-one-out method was chosen to validate this algorithm's robustness (Tsumoto and Hirano, 2014; Wong, 2015). The formula (4) defines the MAE to measure the degree of deviation of the predicted gait trajectory from the real trajectory. The average MAEs of each joint for all subjects and CGA results are presented in Table 1 for the leave-one-out method. For comparison, the mean and SD for each joint are also given, as well as the mean and SDs obtained by the CGA (Cen et al., 2021) methods. The means and SDs of MAEs obtained by GPR are both smaller than those obtained by the CGA (no data of ankle are provided by CGA). This also suggests that the trajectory predicted by GPR is closer to the real trajectory, and the MAEs of different subjects have fewer fluctuations. In Table 2, the means (SDs) of correlation coefficients of each joint for five subjects at different as well as the results from CGA are shown. By comparison, the correlation coefficients obtained by GPR are also better than those obtained by CGA. Therefore, according to the correlation analysis, the IGPG method gives a better prediction with a strong correlation.

**Table 1:** Mean absolute errors (MAEs) of subjects results from Clinical Gait Analysis (CGA) for comparison.

**Joints**	**GPR(deg)**	**CGA(deg)**
Hip(L)	3.36(1.03)	7.66(1.78)
Knee(L)	4.21(1.64)	9.28(3.07)
Ankle(L)	3.35(1.42)	
Hip(R)	3.47(1.18)	7.66(1.78)
Knee(R)	4.51(1.11)	9.28(3.07)
Ankle(R)	3.40(1.25)	

*L: left side; R: right side*.

**Table 2:** Correlation coefficients of subjects and results from CGA for comparison.

**Joints**	**GPR(deg)**	**CGA(deg)**
Hip(L)	0.99(0.01)	0.87(0.07)
Knee(L)	0.97(0.02)	0.85(0.11)
Ankle(L)	0.92(0.04)	
Hip(R)	0.98(0.01)	0.87(0.07)
Knee(R)	0.95(0.02)	0.85(0.11)
Ankle(R)	0.94(0.04)	

*L: left side; R: right side*.

Clinical Gait Analysis is a process of evaluating the locomotion patterns of patients with specific gait-related abnormalities. It is an open-source platform to the public and its data has been uploaded from institutes all over the world. Gait analysis may be executed in a gait analysis laboratory using specialized instruments, such as Vicon Motion Capturing System. This is also referred to as computerized gait analysis, quantitative gait analysis, or CGA. This procedure has been used to understand the etiology of gait abnormalities.

## Subjects and Methods

###  Experiment Criteria

The participants were nine patients (eight men and one woman with mean age=48.22 years) with hemiplegia status-post stroke, who resided in a convalescent rehabilitation ward. All participants had their stroke within 12 months and they had residual right hemiplegia. The demographics of participants are presented in Table 3.

**Table 3:** Participants' demographics (*N* = 9).

**Subject**	**Age**	**Gender**	**Height**	**Weight**	**Paretic**	**FAC**	**Diagnosis**
			**(cm)**	**(kg)**	**side**		
1	42	Male	174	77	Left	IV	The cerebral thrombosis
2	40	Male	170	63	Left	IV	The putamen hemorrhage
3	47	Male	160	58	Right	III	The putamen hemorrhage
4	56	Female	153	51	Right	IV	The putamen hemorrhage
5	65	Male	169	90	Left	III	The cerebral thrombosis
6	40	Male	166	72	Left	IV	The cerebral thrombosis
7	33	Male	165	85	Right	III	The putamen hemorrhage
8	69	Male	165	70	Right	III	The putamen hemorrhage
9	42	Male	168	75	Left	IV	The cerebral thrombosis

Inclusion criteria were as follows:

- First stroke with hemiplegia.
- Functional ambulation category (FAC) of III or IV for the leg.
- Independent or supervision-only walking ability with a quad cane or T cane or no support tool.
- Participants provided written informed consent after the purpose of the study was explained.

Participants were excluded based on the following criteria:

- Unable to understand study-related procedures.
- Exhibited serious hypertension on walking.
- With circulatory disease, respiratory disease, or extreme weakness.
- Failed to receive physical clearance to participate.

###  Experiment Protocol

The experiment was conducted for a total of 2 days.

**Figure 5:**
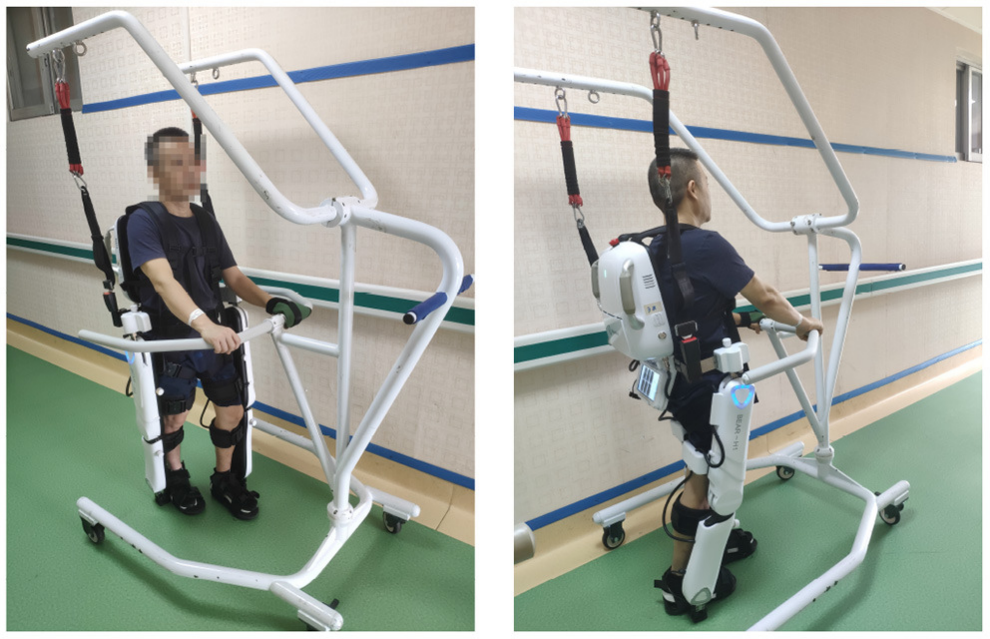
Exoskeleton-robot-assisted walking training.

On the first day of the experiment, 9 patients wore the exoskeleton robot, guided by general gait trajectory which reflects the motion of hip, knee, and ankle joint on healthy people (Murray et al., 1964; Johnston and Smidt, 1969), and received walking training (Figure 5) for 15 min at a fixed frequency.

On the second day of the experiment, the individualized gait which was generalized by our method was applied to the exoskeleton robot to train the same 9 patients with the same method.

**Figure 6:**
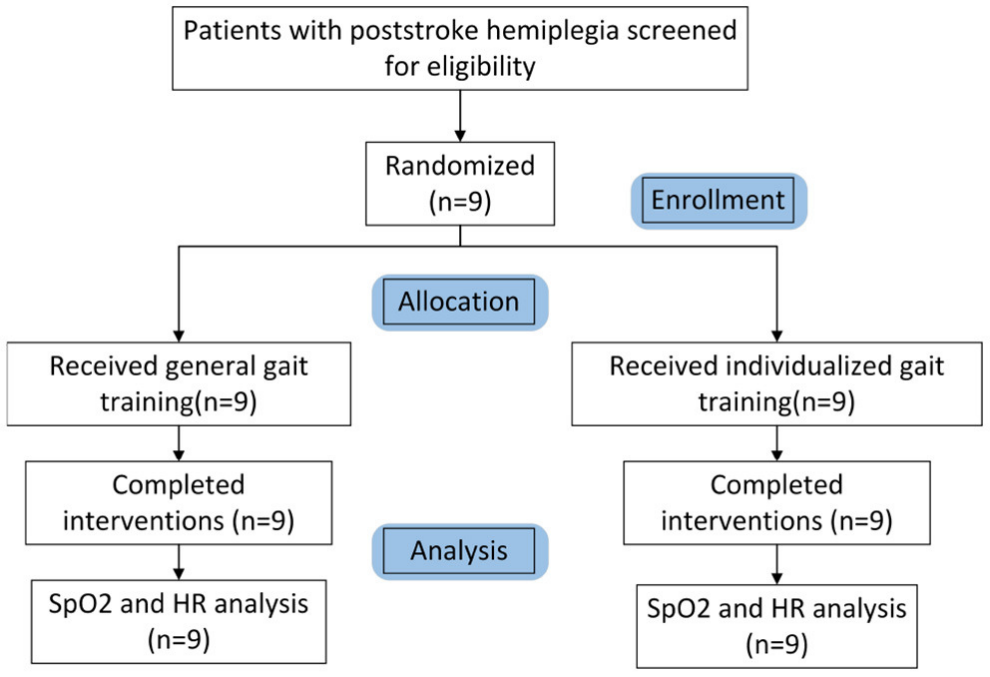
CONSORT participant flow chart.

Dependent variables are HR and SpO2. Dependent variables were sampled four times for each patient: before and after the last individualized-gait exercise treatment, before and after the last general-gait exercise treatment. The effectiveness of the algorithm was verified by comparing patients' decrease of SpO2 and the increase of HR when they were guided by individualized gait trajectory and general gait trajectory, respectively (Figure 6).

The purpose of the experiment is explained to each patient and written informed consents are required to be signed by patients.

## Result

Experiments were administered over 2 days totally, during which the patients were trained by general gait trajectory and individualized gait trajectory sequentially. Results are presented in Tables 4, 5.

**Figure 7:**
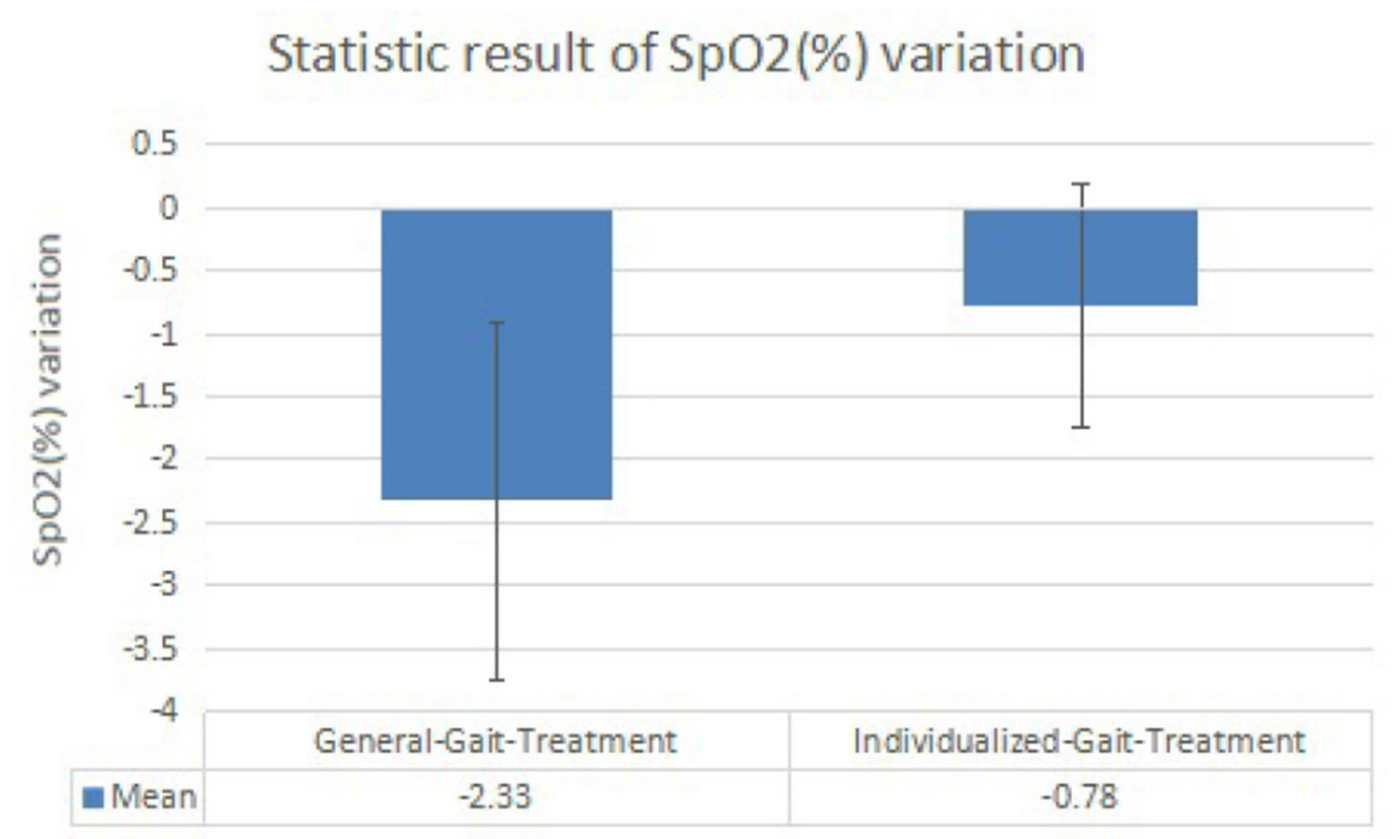
Histogram of peripheral capillary oxygen saturation (SpO2, %) variation results.

**Figure 8:**
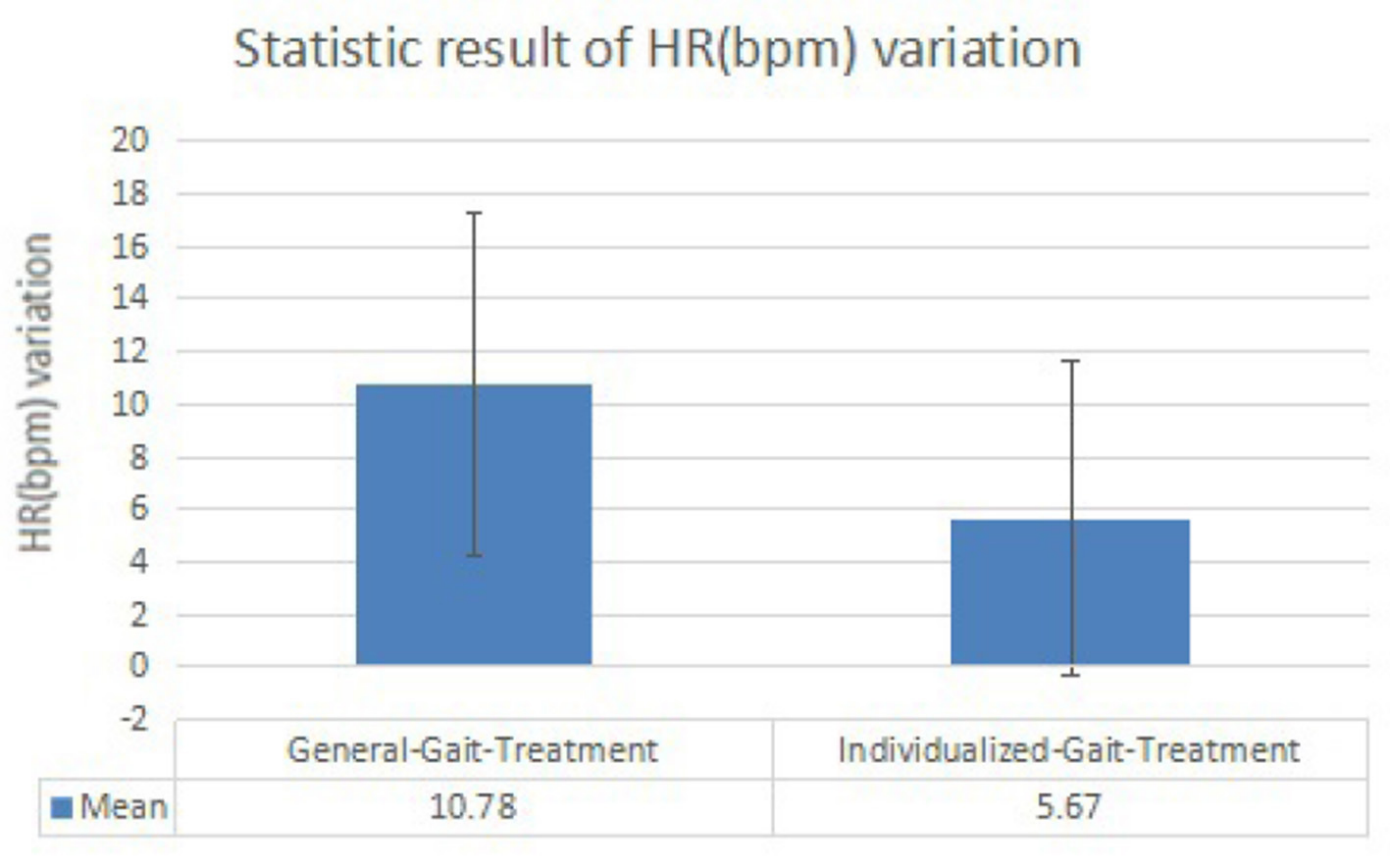
Histogram of heart rate (HR) (bpm) variation results.

- Changes after walking training with exoskeleton robot—we observed changes in SpO2 (Figure 7) and HR (Figure 8) between the before treatment and after treatment in 2 days, respectively. Specifically, changes in HR were more significant. On the contrary, changes in SpO2 were much smaller.
- Differences between two rehabilitation training—from the data collected in the two training sessions, we observed similar varying trends in SpO2 and HR between the before treatment and after treatment. In both groups, the levels of SpO2 decreased and the levels of HR increased (except in rare cases, the levels of HR decreased and the level of SpO2 increased or both remained unchanged. Overall, there was a significant difference in the degree of changes in the levels of SpO2 and HR between two rehabilitation training.) Patients had a smaller SpO2 reduction and larger HR increment when they were guided by the individualized gait trajectory.
- Differences between patients—in both training sessions, the SpO2 levels of different patients before receiving the treatment were roughly the same but started to have slight differences after patients received the individualized treatment, while the HR levels of different patients before and after receiving the treatment was very different. Besides, the degree of dispersion of changes in patients' HR and SpO2 levels under the guidance of two gait trajectories was different. When patients were assisted by the individualized gait trajectories, they had a smaller SD of the changed values of HR and SpO2 levels.

**Table 4:** Results of general-gait-guided treatment.

	**Evaluation items**	**Prior to treatment**	**After treatment**	**variation**
Subject 1	SpO2 (%)	96	91	-5
	HR(bpm)	80	88	8
Subject 2	SpO2 (%)	97	96	–1
	HR(bpm)	82	104	22
Subject 3	SpO2 (%)	98	96	–2
	HR(bpm)	105	120	15
Subject 4	SpO2 (%)	97	96	–1
	HR(bpm)	96	109	13
Subject 5	SpO2 (%)	97	95	-2
	HR(bpm)	82	89	7
Subject 6	SpO2 (%)	98	97	–1
	HR(bpm)	77	89	12
Subject 7	SpO2 (%)	99	96	-3
	HR(bpm)	82	91	9
Subject 8	SpO2 (%)	98	94	–4
	HR(bpm)	76	89	13
Subject 9	SpO2 (%)	98	96	–2
	HR(bpm)	81	79	–2

**Table 5:** Result of individualized-gait-guided treatment.

	**Evaluation items**	**Prior to treatment**	**After treatment**	**variation**
Subject 1	SpO2 (%)	97	95	–2
	HR(bpm)	80	74	–6
Subject 2	SpO2 (%)	96	96	0
	HR(bpm)	90	105	15
Subject 3	SpO2 (%)	97	96	–1
	HR(bpm)	107	113	6
Subject 4	SpO2 (%)	97	98	1
	HR(bpm)	103	113	10
Subject 5	SpO2 (%)	98	97	–1
	HR(bpm)	83	87	4
Subject 6	SpO2 (%)	98	97	–1
	HR(bpm)	75	84	9
Subject 7	SpO2 (%)	99	98	–1
	HR(bpm)	80	88	8
Subject 8	SpO2 (%)	98	96	–2
	HR(bpm)	79	83	4
Subject 9	SpO2 (%)	98	98	0
	HR(bpm)	78	79	1

## Discussion and Conclusions

Lower extremity robotic exoskeleton devices perform the repetitive practice of specific functional tasks in rehabilitation therapy, such as walking training. For each hemiplegic patient, we generalize individualized gaits for their specific training.

This study is to examine the energy consumption effects of individualized gait in walking training among nine patients with hemiplegia status-post stroke with the assistance of a lower-limb exoskeleton robot. HR and peripheral capillary oxygen saturation (SpO2) are selected as the independent variables to reflect energy consumption level (Christensen et al., 1983; Hiilloskorpi et al., 2003). Tables 4, 5 show that SpO2 decreases and HR increases during the walking training. It reveals that our measurement result is acceptable because the internal oxygen is consumed and the heartbeats have a higher frequency to provide blood where the oxygen is stored during the training process. For all patients, the levels of SpO2 are similar but the values of HR are various, showing that the physiological conditions are different among patients. Different HRs are needed to maintain a required blood oxygen level to support their physiological activity. In Table 5, compared to Table 4, the decrease of SpO2 is generally smaller and the HR is also with a tinier change. Tables 6, 7 express the same view precisely. From the aspect of SpO2, the decrease is 2.33% in general-gait-treatment which is larger than the SpO2 level in individualized-gait treatment - 0.78% in average. The HR increases by 10.78 beats per min in general-gait treatment, whereas it is 5.67 beats-per-min growth in individualized-gait treatment. SpO2 and HR are related to the extent of effort the patients made during the walking training period (Fan et al., 2017; Mohan et al., 2017; Nemcova et al., 2020). The more effort they made while walking, the larger proportion of SpO2 decrease and HR increase. As human gaits have specific pattern for each individual, walking with a general gait means the mismatching to original walking habits and therefore patients have to make more effort to overcome the inconformity to follow the gait pattern of the exoskeleton robot. On the other hand, the individualized gait reduces the inconformity between patients and the exoskeleton robot, therefore patients can follow the robot's guidance easier during walking rehabilitation training. Thus, the individualized gait saves energy consumption, and, therefore, the training time for hemiplegic patients can be expanded since more energy remains.

**Table 6:** Statistic result of peripheral capillary oxygen saturation (SpO2) (%) variation.

	**Subject 1**	**Subject 2**	**Subject 3**	**Subject 4**	**Subject 5**	**Subject 6**
General-gait-treatment	–5	–1	–2	–1	–2	-1
Individualized-gait treatment	–2	0	–1	1	–1	–1
	**Subject 7**	**Subject 8**	**Subject 9**	**Mean**	**STD**	
General-gait-treatment	–3	–4	–2	–2.33	1.1414	
Individualized-gait treatment	–1	–2	0	–0.78	0.972	

**Table 7:** Statistic result of heart rate (HR) (%) variation.

	**Subject 1**	**Subject 2**	**Subject 3**	**Subject 4**	**Subject 5**	**Subject 6**
General-gait-treatment	8	22	15	13	7	12
Individualized-gait treatment	-6	15	6	10	4	9
	**Subject 7**	**Subject 8**	**Subject 9**	**Mean**	**STD**	
General-gait-treatment	9	13	–2	10.78	6.553	
Individualized-gait treatment	8	4	1	5.67	5.979	

In the future, more metabolic parameters, i.e., the CO_2_ and O_2_ content in exhaled gas, more patients, and more novel algorithms for gait generalizing will be investigated to find out the different energy consumption situation. A formal clinical will be conducted to verify that the individualized-gait exoskeleton robot has positive effects on rehabilitation for hemiplegic patients.

## Data Availability Statement

The original contributions presented in the study are included in the article/supplementary material, further inquiries can be directed to the corresponding authors.

## Ethics Statement

The studies involving human participants were reviewed and approved by Research Ethics Committee of Qilu Hospital of Shandong University. The patients/participants provided their written informed consent to participate in this study.

## Author Contributions

JY, ZG, and GC made substantial contributions to experimental design. SZ and LX are in charge of data collection, data analysis, and drafting the manuscript. YL, ZZ, and XG offered their expertise advice in screening the subjects, supervising the clinical trial, and interpreting the results. All authors have read and approved the final manuscript.

## Funding

This study was financially supported by Major Scientific and Technological Innovation Projects of Shandong Province under (grant no. 2019JZZY011110), Shenzhen Science and Technology Program (grant no. KQTD20200909114235003), Nanjing Municipal Science and Technology Bureau (grant no. 2019060002), and Wuxi Taihu Talent Plan Medical and Health High-level Talents Project (no. WXTTP2020008).

## Conflict of Interest

JY, GC, and LX are employed by company Shenzhen MileBot Robotics Co., Ltd. The remaining authors declare that the research was conducted in the absence of any commercial or financial relationships that could be construed as a potential conflict of interest.

## Publisher's Note

All claims expressed in this article are solely those of the authors and do not necessarily represent those of their affiliated organizations, or those of the publisher, the editors and the reviewers. Any product that may be evaluated in this article, or claim that may be made by its manufacturer, is not guaranteed or endorsed by the publisher.

## References

[B1] Antonsson E. K. Mann R. W. (1985). The frequency content of gait. J. Biomech. 18, 39–47. 10.1016/0021-9290(85)90043-0 3980487

[B2] Banala S. K. Kim S. H. Agrawal S. K. Scholz J. P. (2008). Robot assisted gait training with active leg exoskeleton (alex). IEEE Trans. Neural Syst. Rehabil. Eng. 17, 2–8. 10.1109/BIOROB.2008.4762885 19211317

[B3] Cen R. Jiang T. Tang P. (2021). Modified gaussian process regression based adaptive control for quadrotors. Aerospace Sci. Technol. 110:106483. 10.1016/j.ast.2020.106483

[B4] Chau T. (2001). A review of analytical techniques for gait data. part 2: neural network and wavelet methods. Gait Posture, 13, 102–120. 10.1016/S0966-6362(00)00095-3 11240358

[B5] Chen B. Ma H. Qin L.-Y. Gao F. Chan K.-M. Law S.-W.. (2016). Recent developments and challenges of lower extremity exoskeletons. J. Orthopaedic Transl. 5, 26–37. 10.1016/j.jot.2015.09.007 30035072 PMC5987051

[B6] Christensen C. C. Frey H. M. Foenstelien E. Aadland E. Refsum H. E. (1983). A critical evaluation of energy expenditure estimates based on individual O_2_ consumption/heart rate curves and average daily heart rate. Am. J. Clin. Nutr. 37, 468–472. 10.1093/ajcn/37.3.468 6829489

[B7] Esquenazi A. Talaty M. Packel A. Saulino M. (2012). The rewalk powered exoskeleton to restore ambulatory function to individuals with thoracic-level motor-complete spinal cord injury. Am. J. Phys. Med. Rehabil. 91, 911–921. 10.1097/PHM.0b013e318269d9a3 23085703

[B8] Fan F. Yan Y. Tang Y. Zhang H. (2017). A motion-tolerant approach for monitoring spo2 and heart rate using photoplethysmography signal with dual frame length processing and multi-classifier fusion. Comput. Biol. Med. 91, 291–305. 10.1016/j.compbiomed.2017.10.017 29102826

[B9] Hartigan C. Kandilakis C. Dalley S. Clausen M. Wilson E. Morrison S.. (2015). Mobility outcomes following five training sessions with a powered exoskeleton. Top. Spinal Cord. Inj. Rehabil. 21, 93–99. 10.1310/sci2102-93 26364278 PMC4568090

[B10] Hiilloskorpi H. Pasanen M. Fogelholm M. Laukkanen R. M. Mänttäri A. (2003). Use of heart rate to predict energy expenditure from low to high activity levels. Int. J. Sports Med. 24, 332–336. 10.1055/s-2003-40701 12868043

[B11] Isola P. Xiao J. Torralba A. Oliva A. (2011). What makes an image memorable? In CVPR 2011, pages 145-152. IEEE. 10.1109/C.V.P.R.2011.5995721

[B12] J Robert Close V. T. I. (1952). The Action of the Ankle Joint. Berkeley, CA: University of California.

[B13] Johnston R. C. Smidt G. L. (1969). Measurement of hip-joint motion during walking: evaluation of an electrogoniometric method. J. Bone Joint. Surg. Am. 51, 1083–1094. 10.2106/00004623-196951060-00003 5805410

[B14] Kagawa T. Ishikawa H. Kato T. Sung C. Uno Y. (2015). Optimization-based motion planning in joint space for walking assistance with wearable robot. IEEE Trans. Rob. 31, 415–424. 10.1109/TRO.2015.2409434

[B15] Kale A. Sundaresan A. Rajagopalan A. Cuntoor N. P. Roy-Chowdhury A. K. Kruger V.. (2004). Identification of humans using gait. IEEE Trans. Image Process. 13, 1163–1173. 10.1109/TIP.2004.832865 15449579

[B16] Khera P. Kumar N. (2020). Role of machine learning in gait analysis: a review. J. Med. Eng. Technol. 44, 441–467. 10.1080/03091902.2020.1822940 33078988

[B17] Kong D. Chen Y. Li N. (2018). Gaussian process regression for tool wear prediction. Mech. Syst. Signal Process. 104, 556–574. 10.1016/j.ymssp.2017.11.021

[B18] Kotwicki T. Walczak A. Szulc A. (2008). Trunk rotation and hip joint range of rotation in adolescent girls with idiopathic scoliosis: does the" dinner plate" turn asymmetrically? Scoliosis 3, 1–11. 10.1186/1748-7161-3-1 18205943 PMC2246099

[B19] Kozlowski A. Bryce T. Dijkers M. (2015). Time and effort required by persons with spinal cord injury to learn to use a powered exoskeleton for assisted walking. Top. Spinal Cord. Inj. Rehabil. 21, 110–121. 10.1310/sci2102-110 26364280 PMC4568092

[B20] Liu M. Wu B. Wang W.-Z. Lee L.-M. Zhang S.-H. Kong L.-Z. (2007). Stroke in china: epidemiology, prevention, and management strategies. Lancet Neurol. 6, 456–464. 10.1016/S1474-4422(07)70004-2 17434100

[B21] Meng W. Liu Q. Zhou Z. Ai Q. Sheng B. Xie S. S. (2015). Recent development of mechanisms and control strategies for robot-assisted lower limb rehabilitation. Mechatronics 31, 132–145. 10.1016/j.mechatronics.2015.04.005

[B22] Meuleman J. Van Asseldonk E. Van Oort G. Rietman H. Van Der Kooij H. (2015). Lopes ii–design and evaluation of an admittance controlled gait training robot with shadow-leg approach. IEEE Trans. Neural Syst. Rehabil. Eng. 24, 352–363. 10.1109/TNSRE.2015.2511448 26731771

[B23] Mohan P. M. Nagarajan V. Nisha A. A. (2017). A frame work to estimate heart rate and arterial oxygen saturation (spo2), in 2017 International Conference on Communication and Signal Processing (ICCSP) (Chennai: IEEE), 1645–1648.

[B24] Morgan K. D. Noehren B. (2018). Identification of knee gait waveform pattern alterations in individuals with patellofemoral pain using fast fourier transform. PLoS ONE 13:e0209015. 10.1371/journal.pone.0209015 30550589 PMC6294430

[B25] Mukaka M. M. (2012). A guide to appropriate use of correlation coefficient in medical research. Malawi Med. J. 24, 69–71. 23638278 PMC3576830

[B26] Mundt M. Thomsen W. Witter T. Koeppe A. David S. Bamer F.. (2020). Prediction of lower limb joint angles and moments during gait using artificial neural networks. Med. Biol. Eng. Comput. 58, 211–225. 10.1007/s11517-019-02061-3 31823114

[B27] Murray M. P. Drought A. B. Kory R. C. (1964). Walking patterns of normal men. J. Bone Joint Surg. Am. 46, 335–360. 10.2106/00004623-196446020-00009 14129683

[B28] Nemcova A. Jordanova I. Varecka M. Smisek R. Marsanova L. Smital L.. (2020). Monitoring of heart rate, blood oxygen saturation, and blood pressure using a smartphone. Biomed. Signal Process. Control 59:101928. 10.1016/j.bspc.2020.101928

[B29] Rajasekaran V. López-Larraz E. Trincado-Alonso F. Aranda J. Montesano L. Del-Ama A. J.. (2018). Volition-adaptive control for gait training using wearable exoskeleton: preliminary tests with incomplete spinal cord injury individuals. J. Neuroeng. Rehabil. 15, 1–15. 10.1186/s12984-017-0345-8 29298691 PMC5751847

[B30] Rasmussen C. E. Williams C. (2006). Gaussian Processes for Machine Learning. Cambridge, MA: The MIT Press.

[B31] Reddy M. K. Rani S. (2016). Statistical image compression using fast fourier coefficients. Int. J. Comput. Appl. 155. 10.5120/ijca201691228217153942

[B32] Riener R. Lünenburger L. Maier I. C. Colombo G. Dietz V. (2010). Locomotor training in subjects with sensori-motor deficits: an overview of the robotic gait orthosis lokomat. J. Healthc. Eng. 1, 197–216. 10.1260/2040-2295.1.2.197

[B33] Santos V. Moreira R. Silva F. (2012). Mechatronic design of a new humanoid robot with hybrid parallel actuation. Int. J. Adv. Rob. Syst. 9, 119. 10.5772/51535

[B34] Stauffer Y. Allemand Y. Bouri M. Fournier J. Clavel R. Métrailler P.. (2008). The walktrainer–a new generation of walking reeducation device combining orthoses and muscle stimulation. IEEE Trans. Neural Syst. Rehabil. Eng. 17, 38–45. 10.1109/TNSRE.2008.2008288 19211322

[B35] Trivino G. Alvarez-Alvarez A. Bailador G. (2010). Application of the computational theory of perceptions to human gait pattern recognition. Pattern Recognit. 43, 2572–2581. 10.1016/j.patcog.2010.01.017

[B36] Tsukahara A. Hasegawa Y. Eguchi K. Sankai Y. (2014). Restoration of gait for spinal cord injury patients using hal with intention estimator for preferable swing speed. IEEE Trans. Neural Syst. Rehabil. Eng. 23, 308–318. 10.1109/TNSRE.2014.2364618 25350933

[B37] Tsumoto S. Hirano S. (2014). Formal analysis of leave-one-out methods based on decremental sampling scheme, in 2014 IEEE/WIC/ACM International Joint Conferences on Web Intelligence (WI) and Intelligent Agent Technologies (IAT), Vol. 2 (San Diego, CA: IEEE), 371–378.

[B38] Vallery H. Van Asseldonk E. H. Buss M. Van Der Kooij H. (2008). Reference trajectory generation for rehabilitation robots: complementary limb motion estimation. IEEE Trans. Neural Syst. Rehabil. Eng. 17, 23–30. 10.1109/TNSRE.2008.2008278 19211320

[B39] Wang L. Tan T. Ning H. Hu W. (2003). Silhouette analysis-based gait recognition for human identification. IEEE Trans. Pattern Anal. Mach. Intell. 25, 1505–1518. 10.1109/TPAMI.2003.125114424592144

[B40] Winter D. A. (2009). Biomechanics and Motor Control of Human Movement. Hoboken, NJ: John Wiley & Sons.

[B41] Wong T.-T. (2015). Performance evaluation of classification algorithms by k-fold and leave-one-out cross validation. Pattern Recognit. 48, 2839–2846. 10.1016/j.patcog.2015.03.009

[B42] Yun Y. Kim H.-C. Shin S. Y. Lee J. Deshpande A. D. Kim C. (2014). Statistical method for prediction of gait kinematics with gaussian process regression. J. Biomech. 47, 186–192. 10.1016/j.jbiomech.2013.09.032 24211221

[B43] Zhang Y. Ma Y. (2019). Application of supervised machine learning algorithms in the classification of sagittal gait patterns of cerebral palsy children with spastic diplegia. Comput. Biol. Med. 106, 33–39. 10.1016/j.compbiomed.2019.01.009 30665140

[B44] Zhang Z. Dong Y. Ni F. Jin M. Liu H. (2015). A method for measurement of absolute angular position and application in a novel electromagnetic encoder system. J. Sens. 2015:503852. 10.1155/2015/503852

